# Survey of public definitions of the term ‘overdiagnosis’ in the UK

**DOI:** 10.1136/bmjopen-2015-010723

**Published:** 2016-04-01

**Authors:** Alex Ghanouni, Susanne F Meisel, Cristina Renzi, Jane Wardle, Jo Waller

**Affiliations:** Health Behaviour Research Centre, Department of Epidemiology and Public Health, University College London, London, UK

**Keywords:** EPIDEMIOLOGY, PUBLIC HEALTH, PREVENTIVE MEDICINE

## Abstract

**Objectives:**

To determine how ‘overdiagnosis’ is currently conceptualised among adults in the UK in light of previous research, which has found that the term is difficult for the public to understand and awareness is low. This study aimed to add to current debates on healthcare in which overdiagnosis is a prominent issue.

**Design:**

An observational, web-based survey was administered by a survey company.

**Setting:**

Participants completed the survey at a time and location of their choosing.

**Participants:**

390 consenting UK adults aged 50–70 years. Quota sampling was used to achieve approximately equal numbers in three categories of education and equal numbers of men and women.

**Primary outcome measures:**

Participants were asked whether they had seen or heard the term ‘overdiagnosis’. If they had, they were then invited to explain in a free-text field what they understood it to mean. If they had not previously encountered it, they were invited to say what they thought it meant. Responses were coded and interpreted using content analysis and descriptive statistics.

**Results:**

Data from 390 participants were analysed. Almost a third (30.0%) of participants reported having previously encountered the term. However, their responses often indicated that they had no knowledge of its meaning. The most prevalent theme consisted of responses related to the diagnosis itself. Subthemes indicated common misconceptions, including an ‘overly negative or complicated diagnosis’, ‘false-positive diagnosis’ or ‘misdiagnosis’. Other recurring themes consisted of responses related to testing (ie, ‘too many tests’), treatment (eg, ‘overtreatment’) and patient psychology (eg, ‘overthinking’). Responses categorised as consistent with ‘overdiagnosis’ (defined as detection of a disease that would not cause symptoms or death) were notably rare (n=10; 2.6%).

**Conclusions:**

Consistent with previous research, public awareness of ‘overdiagnosis’ in the UK is low and its meaning is often misunderstood or misinterpreted.

**Strengths and limitations of this study**

- This is the first study to investigate how the UK general public understands and interprets the term ‘overdiagnosis’.
- Free-text fields allowed participants to respond in their own words without being limited to categories preselected by the researchers.
- The results of this study corroborate those of a previous survey in Australia and are consistent with previous research indicating that overdiagnosis is a difficult concept for people to understand.
- An appreciable proportion of participants' responses were ambiguous in their meaning and some could not be coded into a specific category.

## Introduction

Academics, healthcare professionals and policymakers are becoming increasingly concerned about circumstances in which the health benefits of a given intervention are not considered to clearly justify the associated risks. The *British Medical Journal* has been a prominent voice in this area through their ‘Too Much Medicine’ campaign,^1^ which has recently gained support from the Academy of Medical Royal Colleges.^2^ A central topic within the campaign is ‘overdiagnosis’, defined here as the diagnosis of disease which would never have become clinically apparent in a person's lifetime (ie, causing neither symptoms nor death).^3^ ^4^ Debate continues regarding the magnitude, significance and implications of overdiagnosis. Much attention is focused on breast cancer screening, where estimates of the ratio of screening-prevented breast cancer deaths to overdiagnosed breast cancer cases range from 9:4^5^ to 5:17^3^ for 1000 women screened over 20 years. There is also an increasingly common view that screening invitees should appreciate the possibility of overdiagnosis before deciding whether to participate,^6^ although there is professional controversy regarding how best to inform people about the concept.^7^

Public awareness of overdiagnosis appears to be low in many countries. A study from 2000 in the USA found that only a small minority (7%) of women were aware of the concept of non-progressive breast cancer.^8^ Recently, a focus group study in Korea found that women had difficulty understanding the meaning of overdiagnosis in the context of thyroid cancer screening.^9^ Very similar findings have been reported in a small interview study about breast cancer screening based in Denmark.^10^ In addition, Moynihan and colleagues recently reported a survey of 500 Australian adults drawn from the general population, who were asked to state whether they had seen or heard the term, before indicating what they thought it meant. The majority (50–63%) reported having encountered the term before, but only 41% of the sample were categorised as responding with even an approximately correct definition of the term. Participants categorised as being approximately correct often described it in terms of misdiagnosis, too many diagnoses or overstating the significance of a diagnosis, with 35% of responses falling into one of these categories.^11^ Although these have some similarities with the intended meaning of overdiagnosis, there is an apparent lack of awareness regarding its specific meaning. This suggests that despite attention in health-related literature, overdiagnosis has not become a familiar term among the general public.

The present study extends the previous work by exploring people's definitions of ‘overdiagnosis’ as part of a web-based survey of adults in the UK. We used the same measures of awareness as Moynihan *et al*^11^ to allow comparability between studies.

## Methods

This study took place as part of a web-based survey of attitudes and beliefs about cancer in February 2015. The sample consisted of consenting members of the UK general population aged 50–70 years, recruited through a survey company (Survey Sampling International, SSI). SSI maintains a panel of potential survey participants; these individuals are periodically invited to complete online surveys in exchange for small amounts of compensation (eg, air miles) via a generic recruitment email. The email contained a link that directed participants to the online survey. Quotas were used to ensure an equal proportion of men and women, and equal proportions of participants with different levels of education (three categories: leaving school before 16 years; completing CSEs, O-levels or equivalent; completing A-levels, higher, university education or equivalent).

As in the Australian study, participants were first asked: ‘have you seen or heard the term “overdiagnosis” before today’. Those responding ‘yes’ were then asked ‘what do you understand the term “overdiagnosis” to mean’; those who responded ‘no’ were asked ‘what do you think the term “overdiagnosis” means’.^i^ Participants responded to the latter two questions by completing a free-text field. They were also asked for basic demographic information (gender, age, level of education and ethnicity).

The questions on overdiagnosis formed part of a larger survey on views relating to cancer screening; hence, participants were selected to be approaching or at an age at which they would be eligible for breast or colorectal cancer screening in the UK. Screening-specific questions always came after questions on overdiagnosis, and so responses could not have been influenced by the questions on screening (results not reported here).

Demographic data and self-reported recognition of the term ‘overdiagnosis’ were analysed using simple descriptive statistics. Content analysis was used to examine responses to free-text questions.^12^ Responses were categorised independently by two researchers (AG and SFM), who read participants' answers repeatedly before assigning a provisional code (eg, misdiagnosis) to each one, based on their interpretation. After both researchers had completed their separate coding, the two lists were compared and disagreements resolved through discussion. The final list of codes was then reviewed to determine whether several codes could be meaningfully renamed, grouped together or split into further codes. The overarching themes and specific subthemes identified were then summarised using descriptive statistics and illustrated using exemplar quotes. Responses of participants who reported having seen or heard the term before and those of participants who reported no previous exposure to the term were reported separately.

## Results

### Participant characteristics

The sample comprised 390 adults (201 women; 51.5%) with a median age of 60 years (interquartile range (IQR) 55–65). Most stated their ethnicity as Caucasian (n=382; 97.9%). Per our quota sampling, approximately equal numbers had left school before 16 years (133; 34.1%), completed CSEs, O-levels or equivalent (125; 32.1%), and had completed A-levels, higher, or university degree, or equivalent (132; 33.8%).

### Definitions of ‘overdiagnosis’

One hundred and seventeen participants (30.0%) reported having seen or heard the term previously. Median length of free-text responses relating to participants' understanding was seven words (range 1–42; IQR 4–12.5); for responses from participants who had not seen or heard it before, it was four words (range 1–28; IQR 2–8).

Responses are presented separately for those who had and those who had not previously encountered the term. For both groups, responses could be categorised into one of six broad themes. These are summarised in table 1 (for participants who had previously encountered the term) and table 2 (for those who had not), respectively, along with exemplar quotes. Participants commonly reported either no knowledge of what the term meant (even when they reported having encountered it before) or responded in a way that could not be categorised (frequently because the response was too vague or entirely unclear). Three other themes were aligned with different stages of a patient trajectory: from testing (too many tests) to diagnosis, and treatment. The theme relating to diagnosis was the most prevalent; 38.5% and 26.1% of responses from participants who had and had not been previously exposed to the term were categorised into this theme. Relevant subthemes included an ‘overly negative or complicated diagnosis’, a ‘false-positive diagnosis’ and a ‘misdiagnosis’. Treatment-related responses referred to ‘overtreatment’, ‘inappropriate treatment’ and ‘unbeneficial treatment’. A further broad theme referred to conceptions of how patients' psychology related to overdiagnosis, with the most common subtheme being ‘overthinking’. The final grouping consisted of other responses that could not be categorised into one of the preceding five themes.

**Table 1:** Understanding of ‘overdiagnosis’ among participants who had previously encountered the term (n=117): emerging (sub)themes

Category	Exemplar quote (spelling errors corrected)	n (%)
Response not categorised or no knowledge		
Incoherent, irrelevant or too vague to interpret	“More than a person says.”	21 (17.9)
No knowledge	“Not sure.”	10 (8.5)
Diagnosis-related responses		
Overly negative or complicated diagnosis	“Where certain illnesses are overstated in their severity.”	12 (10.3)
False-positive diagnosis	“Diagnosis of a condition that is not really there.”	11 (9.4)
Overdiagnosis	“I think it means the diagnosis of a disease that will not cause symptoms or death.”	9 (7.7)
Too many diagnoses	“Finding out all the complaints that may be wrong with you.”	8 (6.8)
Overly detailed diagnosis	“A condition has been over-described.”	4 (3.4)
Misdiagnosis	“Diagnosing minor problems rather than the real problem at source.”	1 (0.9)
Diagnosis based on stereotypes	“Diagnosis based on stereotypes e.g. The patient is overweight therefore he will have a CVA.”	1 (0.9)
Test-related responses		
Too many tests	“Patient undergoing more tests than necessary to diagnose a disease.”	6 (5.1)
Treatment-related responses		
Inappropriate treatment	“Taking medicine not needed.”	1 (0.9)
Overtreatment	“Had taken too many pills, or dr. prescribe too many different pills the person must take, when don’t need.”	1 (0.9)
Responses related to patients' psychology		
Overthinking	“When you think things through to such an extent that you confuse things or make them more complicated or important than they deserve to be.”	20 (17.1)
Overly health-sensitive	“Over-analysing of minor conditions until you are convinced that there is a condition to be treated.”	4 (3.4)
Anticipating a worse diagnosis	“Presuming something nasty is wrong before running tests.”	3 (2.6)
Other responses		
Medicalising issues unrelated to health	“When non-health-related issues are medicalised.”	2 (1.7)
Defensive medicine	“Must have an answer, doctors not wanted to be sued.”	1 (0.9)
Iatrogenic illness	“Iatrogenic illness”	1 (0.9)
Too many attempts at prevention	“Too much preventing what may happen or not.”	1 (0.9)

**Table 2:** Interpretation of ‘overdiagnosis’ among participants who had not previously encountered the term (n=273): emerging (sub-)themes

Category	Exemplar quote (spelling errors corrected)	n (%)
Response not categorised or no knowledge		
No knowledge	“Don't know.”	103 (37.7)
Incoherent, irrelevant or too vague to categorise	“Going over the top.”	44 (16.1)
Diagnosis-related responses		
Overly negative or complicated diagnosis	“Diagnosing a disease to a worse state than actual.”	30 (11.0)
False-positive diagnosis	“It sounds like being told you have things you don’t have.”	13 (4.8)
Misdiagnosis	“The wrong diagnosis of an illness.”	12 (4.4)
Overly detailed diagnosis	“Too much information on a probable problem.”	9 (3.3)
Too many diagnoses	“Too many different diagnosis from various medical professionals.”	6 (2.2)
Overdiagnosis	“Making People Sick in the Pursuit of Health.”	1 (0.4)
Test-related responses		
Too many tests	“Too many health checks.”	6 (2.2)
Treatment-related responses		
Overtreatment	“Treating an illness in a stronger way than necessary.”	6 (2.2)
Inappropriate treatment	“Wrong treatment or pills.”	2 (0.7)
Unbeneficial treatment	“Giving medical assistance when there is no hope.”	1 (0.4)
Responses related to patients’ psychology		
Overthinking	“Complicating a problem by thinking too much about it.”	15 (5.5)
Overly health-sensitive	“Worrying too much about health issues, continually seeking explanations.”	10 (3.7)
Anticipating a worse diagnosis	“Looking at worst case scenario before having all the facts.”	8 (2.9)
Other responses		
Multiple (medical) opinions	“Too many people involved in a medical decision.”	5 (1.8)
Defensive medicine	“Treatment, just to be on the safe side.”	2 (0.7)

Responses were rarely consistent with ‘overdiagnosis’ itself, whether or not participants had previously been exposed to the term (n=9, 7.7%; n=1, 0.4%). All such responses are reported in box 1.

Box 1 **All responses coded as consistent with ‘overdiagnosis’**

Response (spelling errors corrected)

Participants reporting having previously seen or heard the term

“An example would be with some forms of health screening you get a positive result which leads to further investigation and treatment for a condition which would probably never have killed you. There is a type of breast cancer (DCIS) which falls [response was truncated by survey character limit].”

“Treating conditions like tiny lesions in the breast that may have resolved themselves without treatment”

“I think it means the diagnosis of a disease that will not cause symptoms or death.”

“Yes, it is the diagnosis of a disease that will not cause the death of a patient.”

“Reacting too much to minor and potentially insignificant things found during an investigation, with the result that they are treated unnecessarily.”

“When you are screened for something it is a false positive or a positive on something that would not harm you in your likely lifespan.”

“A diagnosis of a disease that will never happen or cause a problem.”

“Some people can be diagnosed and have unsuitable or unnecessary treatment.”

“Overdiagnosis is the diagnosis of disease that will never cause symptoms or death during a patient's lifetime.”

Participants reporting no previous exposure to the term

“Making People Sick in the Pursuit of Health.”

## Discussion

This survey of definitions of ‘overdiagnosis’ among adults in the UK found that despite approximately one in three people stating that they had seen or heard the term before, only a minority (2.6% of all participants) provided a response that was even broadly consistent with the meaning considered correct: diagnosis of a disease that would not cause symptoms or death.^3^ This finding can be interpreted in light of the inconsistent usage of the term among academics and clinicians. As Carter *et al*^4^ note, the intended meaning can vary, depending on the specific context being addressed within the general domain of ‘Too Much Medicine’. For example, the term may be used to describe detection of ‘incidentalomas’ in lung cancer screening,^13^ or diagnosis due to diagnostic criteria being expanded (eg, pre-diabetes^14^ and attention-deficit/hyperactivity disorder^15^). Hence, it is not surprising that a single clear meaning has not reached public awareness. However, concerns regarding overdiagnosis are likely to be an increasingly influential aspect of both policy-level and patient-level decisions. Consequently, policymakers, healthcare providers and communicators may consider this to be an important concept that the public should understand. One risk of a lack of awareness is scepticism regarding the true purpose of an attempt to reduce the delivery healthcare interventions (eg, the belief that it is an attempt to reduce costs). This may account for the generally negative views among women regarding the US Preventive Services Task Force (USPSTF) recommendation that women aged 40–49 should not undergo routine mammography screening^16^ ^17^ and it might be hypothesised that there are similar negative perceptions among men regarding the USPSTF recommendation against prostate-specific antigen screening in 2012.^18^ The present findings indicate that there is substantial potential to increase public awareness.

After excluding responses that could not be categorised and those where participants stated they had no knowledge, the most commonly occurring themes were related to diagnosis, including an ‘overly negative or complicated diagnosis’, or a ‘false-positive diagnosis’. These findings are similar to those of the Australian survey,^11^ in which 22% thought it meant exaggerating an existing condition and 10% thought it meant diagnosing a non-existent condition. The patient-related subthemes such as ‘overthinking’ were more surprising, showing that patients do not necessarily only consider the healthcare provider's or the healthcare system's responsibility in influencing overdiagnosis, but also recognise the possible influence of patient attitudes towards health and illness. Some previous studies^19^ have provided participants with information that aims to clarify the distinction between overdiagnosis and false positives; the present findings suggest that there may be additional assumptions to be addressed.

Our findings are also consistent with discussion group studies in the UK and Australia where the term ‘overdiagnosis’ was explained to lay members of the public in the context of breast cancer screening. These studies reported that most participants found the term counterintuitive, in part because it involved understanding that some medical conditions, including cancer, will never cause harm.^20^ ^21^ The term was also seen as difficult for participants to understand. Participants were given detailed information on overdiagnosis using a method that allowed them to ask and receive answers to questions that arose. However, when asked a multiple choice question on the most applicable definition, 16% gave an incorrect answer and a further 24% gave only a partially correct answer.^20^ Comparable trends have also been seen in both quantitative^8^ and qualitative studies^9^ ^10^ from other countries, which have reported both low levels of awareness and difficulty understanding the term.

This study has limitations. Coding of participants' definitions necessarily relied on the authors' interpretations of brief free-text responses, and so the reader may disagree with some of our coding. Furthermore, as previously noted, there is continuing academic debate regarding the most appropriate definition of overdiagnosis.^4^ ^22^ In addition, some responses coded as consistent with overdiagnosis also contained misconceptions (eg, relating to false positives). In these cases, the most appropriate code to use was ambiguous. Similarly, the brevity of participants' responses precluded an in-depth understanding of their intended meanings, resulting in categories that warrant further exploration. In particular, participants' ideas regarding ‘overthinking’ may have been related to the source and context in which they encountered the term but these factors were not recorded in the present study. There was also an appreciable proportion of responses that could not be coded. Future qualitative research could be undertaken to address this. Future research could also evaluate whether alternative terminology (eg, overdetection)^19^ would be more intuitive to participants, resulting in a greater proportion of correct interpretations. Finally, survey response rates were not available from the survey company, creating uncertainty regarding the representativeness of the sample.

In conclusion, this study found that ‘overdiagnosis’ was rarely defined correctly by the public, indicating substantial scope to increase awareness. Future research should be designed with an assumption of extremely low pre-existing knowledge of the concept in the general population.

## Footnotes

i An error in the skip logic of the survey meant that participants who responded ‘yes’ were also asked the second question. Hence, data on questions asked in error were not analysed since participants often responded with the same answer or noted that they had already answered a very similar question. One participant who responded ‘no’ was able to complete both questions and so was excluded from the analysis since their answer to the second (correctly asked) question may have been affected by the first (incorrectly asked) question.
